# Knowledge, attitudes, and support of women's menstrual experiences: A cross-sectional survey of men in Kampala, Uganda

**DOI:** 10.1016/j.healthplace.2025.103439

**Published:** 2025-05

**Authors:** Madeleine Patrick, Nicole Stephan, Thea Mink, Tanushree Bhan, Barbra Mary Aine, Nabutuwa Viola Matanda, Amelia Conrad, Sheela S. Sinharoy, Bethany A. Caruso

**Affiliations:** a Hubert Department of Global Health, Rollins School of Public Health, Emory University, Atlanta, USA; b City Wide Inclusive Sanitation, Kampala Capital City Authority, Kampala, Uganda

## Abstract

Menstruation has received growing attention in public health research, particularly among adolescents in schools. However, fewer studies have engaged adult men. This secondary analysis (1) assessed alignment between men's perceptions of women's menstruation practices and women's actual practices; 2) examined associations between men's demographic characteristics and their perception of menstruation-related communication norms, and 3) assessed if men's perceptions of these norms are associated with their support of menstruating women or girls in their household.

Cross-sectional data were collected via household survey from men (n = 344) and women (n = 297) in Kampala, Uganda in 2022. Outcomes were two norms questions on the appropriateness of discussing menstruation in public and in front of men. We used a combination of Poisson and Firth's regressions. Presence of a menstruator in the household was positively associated with men's perception that it is acceptable to discuss menstruation in front of men (PR: 1.37; 95% CI: 1.07, 1.76; p < 0.01) or in public (PR: 1.12, 95% CI: 1.12, 2.46; p < 0.01). Supportive norms were associated with supportive behaviors; men who agreed that women may discuss menstruation in public and in front of men were more likely to report a willingness to talk to women about menstruation-related problems (PR = 1.75, 95% CI: 1.38, 2.22; p < 0.001). Our findings support the need for norms change to reduce stigma around discussing menstruation. Programs aiming to improve menstrual health should consider men's knowledge of menstruation, their role in the household environment around menstruation, and how norms may contribute to how they support menstruators in their households.

## Highlights

• Men should be engaged in menstruation education and programming.
• Men lack knowledge of women's menstrual practices, such as materials and disposal.
• Men report more supportive norms around discussing menstruation than women.
• Living with a menstruator is linked to perception of supportive norms among men.
• Reporting supportive norms is directly linked to providing support for menstruators.

## 1 Introduction

Approximately 1.8 billion individuals of reproductive age worldwide experience menstruation, yet menstrual health and hygiene needs often go unmet. (UNICEF) There has been greater acknowledgement in recent years of the importance of menstrual health for human rights, gender equality, and progress across the Sustainable Development Goals (Olson et al., 2022; Sommer et al., 2017, 2021; Winkler and Roaf, 2014). As recently as June 2024, the 10.13039/100004420United Nations Human Rights Council adopted a resolution highlighting the role of menstruation in health and gender equality (Menstrual health is a fundamental, 2024). Despite this increased attention, menstruation remains a stigmatized and often taboo topic in both high- and low-income countries(Barrington et al., 2021; Hennegan et al., 2019; Caruso et al., 2019), contributing to gender inequality (Wilson et al., 2018). These menstrual taboos and accompanying negative social norms may prevent those who menstruate from having access to physical resources, like menstrual materials, and social resources, like support from family members, needed for maintaining adequate menstrual health (Barrington et al., 2021; Hennegan et al., 2019).

Menstrual health, defined as “a state of complete physical, mental, and social well-being and not merely the absence of disease and infirmity, in relation to the menstrual cycle,” requires not only access to materials, but also to adequate sanitation facilities and a supportive and safe social environment (MacRae et al., 2019; Hennegan et al., 2021; Schmitt et al., 2018). For a sanitation facility to be ‘female-friendly' and to adequately enable menstrual health, it must be safe and private, have soap and water for cleaning the body and/or menstrual materials as well as disposal options for menstrual materials (MacRae et al., 2019; Hennegan et al., 2021; Schmitt et al., 2018). While these resources are noted to be important primarily for women and girls, particularly those who menstruate, qualitative studies in India have noted that men often have the decision-making power to allocate household resources for menstrual products and make decisions regarding household sanitation facilities (Routray et al., 2017; Mahon et al., 2015). Due to the power imbalances in those environments, men are critical for enabling a supportive environment for menstrual health and hygiene (Mahon et al., 2015).

Despite the importance of men's role in the family, there are limited data on adult men's understanding of women's menstrual practices and experiences, and little knowledge on men's perceptions of norms around menstruation, particularly in low- and middle-income countries (Erchull, 2020). Most studies engaging males have been at the school-level, assessing knowledge and attitudes toward menstruation among boys or impacts on girls' absenteeism and other outcomes as a result of menstrual interventions targeting boys (Kansiime et al., 2020, Khan et al., 2023). A qualitative study among schoolboys in India found that boys often learned about menstruation by overhearing conversations or piecing together observations of cultural rituals like seclusion, not from formal education or direct conversations with women or girls; boys were interested in learning more and sympathetic to their menstruating sisters and classmates, despite having gleaned a relatively negative impression of menstruation (Mason et al., 2017). Similarly, a mixed-method study in rural Zambia noted that boys were curious about menstruation in focus group discussions, but boys' knowledge was centered on menstrual restrictions for women and both mothers and boys stated that parents do not discuss menstruation in the presence of boys (Shah et al., 2019), In rural northern Tanzania, a survey of four schools found that 84% of schoolboys would like to have someone they could ask questions about menstruation, even though a large majority of boys thought it was inappropriate for girls to discuss menstruation with males, including their fathers (Benshaul-Tolonen et al., 2020). However, while several studies indicate that boys are curious about menstruation and open to learning more, there are few studies on adult males or on social environments outside the school such as households. Qualitative studies carried out with men in India and Taiwan have shown that men do not receive menstrual education, either formally or informally (Mahon et al., 2015; Chang et al., 2012; Mason et al., 2017). In a systematic review of women's experiences in high-income countries, few studies found that men were sources of menstrual support or education compared to women in the family (Barrington et al., 2021). Because of stigma around menstruation, women and girls have been discouraged from talking about menstruation in mixed-gender settings, meaning that men and boys may not be involved in discussions about menstruation that occur in the home or in schools (Chang et al., 2012).

There is a need for greater understanding of men's perceptions of menstruation and if and how these perceptions influence both women's practices and experiences and men's behaviors in support of those who menstruate in their households. To address these gaps, the goal of this study is to investigate men's perceptions and perspectives of menstruation in Kampala, Uganda. Specifically, this study aims to: 1) determine if men's perceptions of women's menstruation practices are aligned with women's actual practices; 2) examine associations between men's demographic characteristics and their perception of community norms related to communication about menstruation, and 3) assess if men's perceptions of norms around menstruation-related communication are associated with their provision of meaningful support for menstruating women and girls (MWG) in their household.

## 2 Methods

### 2.1 Study design

This study is a secondary analysis of a subset of cross-sectional data from the Measuring Urban Sanitation and Empowerment (MUSE) project, which aimed to develop and validate the Agency, Resources, and Institutional Structures for Sanitation-related Empowerment (ARISE) scales to measure women's empowerment related to sanitation in urban areas of low- and middle-income countries (Sinharoy et al., 2024). The resulting scales included modules specific to menstruation to be delivered alongside or independently of the sanitation modules.

This secondary analysis uses data from Kampala, where one of the partners in this research, the Kampala Capital City Authority (KCCA), was interested in men's perceptions of menstruation. Emory University collaborated with KCCA to adapt survey prompts from the existing survey designed for women to be suitable to deliver to men. The survey delivered to men therefore included many of the survey prompts originally written for women as well as additional questions added or adapted for men. A total of three surveys were delivered: a long-form survey for women, a short-form survey for women, and a survey for men. This study includes data collected from the long-form survey for women and the survey for men.

### 2.2 Study setting, eligibility, and recruitment

Data were collected from both women and men. We collected data from women in each of Kampala's five administrative divisions (Central, Kawempe, Makindye, Nakawa, and Rubaga); data were collected from purposively selected parishes within each division, which were identified for variation in income and sanitation access. We collected data from men in a subset of six parishes in three of the five divisions (Central, Kawempe, and Rubaga). This analysis uses data *only* from parishes where data were collected from both women and men, all of which were identified as slums by KCCA.

The target sample size for MUSE was 700 women per city based on standard guidelines for scale development (Sinharoy et al., 2022a; DeVellis and Thorpe, 2021). Sample size per parish was proportionate to the number of households per parish, resulting in a planned sample of 285 women in the six parishes included in this study. Because men were not the focus of the parent study, the target sample size of 300 for men was chosen based on available resources.

Eligible participants were men or women who were residents of Kampala, age 18 or older, able to communicate in English or Luganda, and able to demonstrate understanding of the study purpose and consent form. Questions about menstruation practices or behaviors were delivered only to women who had experienced a menstrual period in the year prior or men who reported living with a menstruating women or girl (MWG). In this paper, menstruating women or girls will be referred to as MWG for the sake of brevity.

Women were recruited by following a simple random-walk sampling method, wherein female enumerators walked in pairs through selected neighborhoods, with each enumerator knocking on every third door on opposite sides of the street from each other. At the end of each survey, we asked women whether they would allow us to recruit a man from their household to take another a survey to enable comparison of intrahousehold dyads; if they agreed, a male enumerator returned to the house to survey a man. However, it was challenging to recruit men from those households due to difficulty scheduling and because many participating women did not give permission to recruit men in their households. We were able to collect data from only ten dyads. We therefore also recruited men from households where a corresponding woman was not surveyed by following the same random sampling method used for women (Sinharoy et al., 2024).

### 2.3 Data collection

Data were collected from April 11–29, 2022, by enumerators from CME Solution Ltd, a local consultancy firm hired by Athena Infonomics to conduct data collection, using tablets equipped with Kobo Toolbox. There were separate survey tools for men and women; women were trained to collect data from women, and men were trained to collect data from men. Athena Infonomics coded Kobo Toolbox with the surveys. Field supervisors from CME verified the completion of surveys after each day of data collection and uploaded data to a secure data storage platform.

### 2.4 Measures

All participants were asked the same survey questions on demographic characteristics and norms around discussing menstruation (Sinharoy et al., 2023, 2024; Caruso et al., 2022). Women who experienced a menstrual period in the year prior were asked additional questions on menstruation practices. Men who live with a MWG were asked questions on their perceptions of women's menstrual practices. They were also asked questions on if they provide support for MWG in their household. Menstruating women were asked questions on the level of support they experience in their household; however, because the questions ask about support from anyone in their household as opposed to men, we have not included those items in this analysis.

#### 2.4.1 Aim 1: descriptive exploration of (a) menstrual practices and (b) norms

**Fig. 1:**
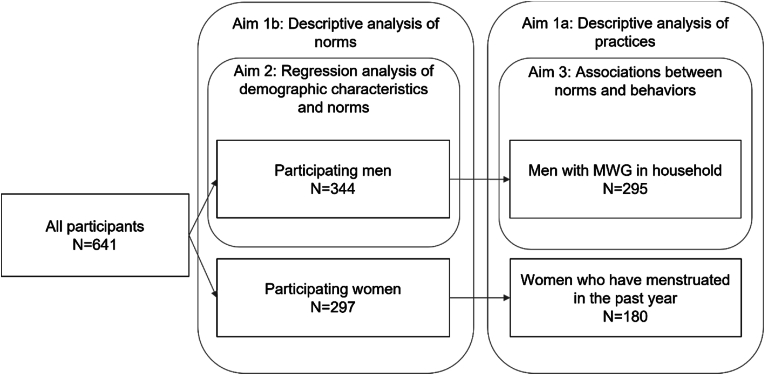
Analytic sample by aim.

To determine if men's perceptions of women's menstruation practices are aligned with women's actual practices (aim 1), we asked women about their practices and men what they thought the practices of MWG in their household were. We created an analytic dataset that included data from men who lived with a MWG and women who had experienced a menstrual period in the prior year (Fig. 1). Using that dataset, we calculated descriptive statistics to understand the distribution of men's and women's responses to questions related to women's menstrual practices (Fig. 1). Because we were unable to collect data among household dyads in sufficient numbers for analysis, the descriptive analyses are treated as reflective of community-level normative practices, rather than a direct comparison of men's knowledge of the practices of MWG in their household. To see if women and men's perceptions of supportive norms related to communication around menstruation were aligned, we also calculated descriptive statistics on the full dataset of men and women, regardless of presence of MWG in the household for men or recent menstruation for women, for two individual items from the ARISE scales: “In this community, it is appropriate for women to discuss menstruation-related issues publicly” and “In this community, it is appropriate for women to discuss menstruation-related issues in front of men” (Fig. 1). Participants were asked to state their level of agreement with each item using a Likert scale of: Strongly disagree, Disagree, Agree, and Strongly agree. Responses were then recoded into either Agree (1) (those who responded with Agree or Strongly Agree) and Disagree (0) (those who responded with Disagree or Strongly Disagree).

#### 2.4.2 Aim 2: regression analysis of demographic characteristics and norms

For our second aim of examining men's demographic characteristics and the perception of supportive norms around menstruation, we used the full men's dataset (Fig. 1). The outcomes (men's perceptions of supportive norms around menstruation) were measured using the same two norms items as in aim 1, capturing if it is appropriate for women to discuss menstruation publicly and in front of men. Response options were again grouped into Agree (1) or Disagree (0). The exposures were measured using demographic questions on age, education (primary or less education vs. more than primary education), ethnicity (Baganda vs. other), religion (Catholic, Protestant, Muslim, or other), and the presence of a MWG in the home. Ethnicity was grouped into Baganda and “other” due to small sample sizes across ethnicity response options (Table 1).

**Table 1:** Demographic characteristics of participants.

Characteristic	Men		Women	
	N = 344	%	N = 297	%
**Age in years (mean (SD))**	34.8	SD: 12.1	33.5	SD: 12.5
**Education**^a^	344		290	
Primary or less	54	15.7%	118	39.9%
Secondary	166	48.3%	136	45.9%
Post-secondary	124	36.0%	42	14.2%
**Marital Status**^b^	343		296	
Single/never married	129	37.6%	59	19.9%
Married	133	38.8%	69	23.3%
Unmarried, living with partner	58	16.9%	99	33.4%
Divorced or separated	17	5.0%	48	16.2%
Widowed	6	1.7%	21	7.1%
**Ethnicity**	344		295	
Acholi	5	1.5%	3	1.0%
Alur	1	0.3%	1	0.3%
Baganda	169	48.1%	123	41.4%
Bagisu	9	2.6%	11	3.7%
Bakiga	16	4.7%	16	5.4%
Bakonzo	6	1.7%	2	0.7%
Banyankore	46	13.4%	51	17.2%
Banyoro	13	2.8%	12	4.0%
Basoga	12	3.5%	23	7.7%
Batoro	6	1.7%	19	6.4%
Iteso	9	2.6%	2	0.7%
Lango	10	2.9%	4	1.3%
Lugbara	5	1.5%	2	0.7%
Nubian	1	0.3%	0	0.0%
Other	36	0.0%	26	8.8%
**Religion**^c^	344		296	
Christian (Catholic)	106	30.8%	89	30.1%
Christian (Protestant	172	50.0%	145	49.0%
Muslim	50	14.5%	51	17.2%
Other	16	4.7%	11	3.7%
**Menstruation status**^c^**♀**			296	
Has experienced a menstrual period in the last year	–	–	180	60.8%
**Lives with menstruating woman or girl**^d^**♂**	342			
Yes	295	57.0%	–	–
No	123	36.0%	–	–
Don't know	66	6.4%	–	–
Don't know what menstruation is	4	0.6%	–	–
Part of household dyad**♂**	342			
Yes	10	2.9%		
Part of household dyad and lives with MWG**♂**	10			
Yes	5	50.0%	–	–

**♂** Indicates that an item was delivered to men only.

♀ Indicates that an item was delivered to women only.

a One woman selected “choose not to answer.”

b One man and one woman selected “choose not to answer.”

c One woman selected “choose not to answer.”

d Two men selected “choose not to answer.”

We conducted a Poisson regression analysis with robust standard errors to evaluate the associations between men's demographic characteristics and their perception of norms. Poisson regression with robust standard errors provides a valid alternative for estimating prevalence ratios in cross-sectional studies, offering stable estimates while maintaining interpretability (Coutinho et al., 2008; Deddens and Petersen, 2008; Espelt et al., 2016). The exposure variables representing demographic characteristics were selected a priori based on existing literature (Douglas, 2003; Kamat and Tharakan, 2021; Tan et al., 2017; Kvalem et al., 2024; Siddique et al., 2023). In addition to the demographic characteristic exposures, neighborhood was assessed as a potential confounder because norms questions specifically ask about appropriateness within the participant's community. Therefore, we anticipated that location may influence both men's agreement with menstruation norms and their behaviors toward menstruating women. A confounding assessment was performed, and neighborhood was included in the final model if the adjusted prevalence or odds ratios differed by more than 10% from the crude ratios (Inoue et al., 2024; Skelly et al., 2012). All unadjusted and adjusted results can be found in Supplementary Table B.

#### 2.4.3 Aim 3: regression analysis of norms and behaviors

To determine if men's perception of supportive norms was associated with meaningful support for MWG in their households, we used the analytic dataset of men with a MWG in their household (Fig. 1). The outcomes were measured using the following questions: “Women in my household may be scolded or punished if they speak up about menstruation-related sanitation concerns or problems,”; “Women in my family can freely talk about problems related to menstruation with me”; and “I provide money for women in my household meet their menstrual needs.” The exposures were measured using the same norms items capturing if it is appropriate for women to discuss menstruation publicly and in front of men (as in Aim 1 and 2). We recategorized the data into men who agreed with at least one of the two norms, men who agreed with both supportive norms, and men who agreed with neither.

We conducted Poisson regression analyses with robust standard errors to evaluate two sets of associations between men's perception of norms and meaningful support. One supportive menstruation practice, “I provide money for women in my household to meet their menstrual needs,” was a rare outcome, with only 7% of the sample agreeing with the practice. As such, Firth's logistic regression was used to mitigate bias resulting from rare events and ensure that models would converge (Xue et al., 2017). As was done for Aim 2, neighborhood was assessed as a potential confounder. All unadjusted and adjusted results can be found in Supplementary Table C.

Data were cleaned using Stata (version 18.0) (StataCorp, 2021). All analyses were conducted using R Studio version 2024.04.2 (RStudio Team, 2024). For each model under Aim 2 and Aim 3, prevalence ratios (PR) or odds ratios (OR) with 95% confidence intervals (CIs) and Wald p-values were reported.

### 2.5 A note on inclusivity

This study recruited men and women, and the data collection instrument uses the language of “women” and “girls” throughout. When recruiting, we asked to speak with a “man” or “woman” at the household level; we did not recruit specifically for menstrual status and we did not ask further questions regarding gender identity or sex. It is therefore possible that we missed opportunities to collect data from menstruators who do not identify as women; there are ethical challenges in doing so since attempting to engage these individuals for a survey could result in unintended harm. Because of the methods and language in the survey tool used, we use the terms “men,” “women,” and “girls” throughout the methods and results section. When incorporating literature, we have used the language consistent with the referenced study.

### 2.6 Ethics and informed consent

Study protocols were reviewed and approved by ethics committees at Emory University in Atlanta, GA, USA (IRB00110271) and Makerere University (MAK-SHSREC Ref. No. 2019-038). Participants provided informed signed consent before participating in surveys and were compensated UGX 10,000 (USD 2.70).

## 3 Results

### 3.1 Participant demographic characteristics

A total of 344 men and 297 women completed the survey (Table 1). The average age of participating men was 34.8 (range: 18–86) years old and the average age of participating women was 33.5 (range: 18–80) years old. 46% of men and 56% of women were married (men: 39%; women: 23%) or living with a partner (men: 17%, women: 33%). Among men, 16% had received a primary education or less, and among women, 40% had received a primary education or less. The predominant ethnic group among men and women was Baganda (men: 48%; women: 41%), followed by Banyankole (men: 13%; women: 17%). Half of participants practiced Protestant Christianity (men: 50%; women: 49%), almost a third practiced Catholic Christianity (men: 31%; women: 30%), and 15% of men and 17% of women practiced Islam (Table 1). Of participating men, 57% lived with a menstruating woman or girl, and of the participating women, 61% had menstruated in the year prior to data collection.

### 3.2 Aim 1: descriptive statistics on menstrual practices (reported and perceived) and on norms around discussing menstruation

Among the 318 men who provided a response, 61% reported there to be a woman or girl in their household who had menstruated in the last year (MWG). Over half of the women respondents (61%) had experienced a menstrual period in the past year. Only 3% of participating men with MWG were part of household dyads where data was also collected from menstruating woman; therefore, the results should be interpreted as a presentation of normative behaviors and assumptions in the community.

We observed differences between women's responses about their menstrual practices and men's responses about what they believe MWG in their household do (Table 2). Among the 180 women, the most frequently reported material used for menstruation was single-use/disposable pads (76%); 82% of men also responded that single-use/disposable pads were the material used most often by the MWG in their household. While no men (0%) reported that MWG in their household used cloth for menstrual materials, 10% of women reported that they used cloth. Similarly, only 4% of men reported that MWG in their household used reusable pads while 13% of women reported using reusable pads.

**Table 2:** Menstrual practices of women, as reported by men who live with MWG and by women who had experienced a menstrual period in the prior year.

Characteristic	Men N = 195		Women N = 180	
	N	%	N	%
**Menstrual materials used most often**^a^				
Cloth	0	0.0%	19	9.5%
Reusable sanitary pads	7	3.6%	27	13.4%
Single-use/disposable sanitary pads	160	82.1%	152	75.6%
Cotton wool	1	0.5%	2	1.0%
Underwear alone	0	0.0%	1	0.5%
Other	27	13.8%	–	–
**Location where menstrual materials are changed**				
Private toilet inside house	10	5.1%	8	4.4%
Bathroom (bathing room) inside house	13	6.7%	3	1.7%
Other room inside house	103	52.8%	66	36.7%
Private toilet outside house	6	3.1%	36	20.0%
Shared or public toilet	2	1.0%	21	11.7%
Private bath (bathing) room outside my house	7	3.6%	37	20.6%
Don't know	54	27.7%	–	–
Other	–	–	9	5.0%
**How materials are disposed of**^b^				
Flushed/put in pit latrine	6	3.1%	37	20.6%
Put in rubbish bin	95	48.7%	105	58.3%
Thrown in open drain	3	1.5%	1	0.6%
Burned	19	9.7%	13	7.2%
Do not dispose	2	1.0%	21	11.7%
Don't know	69	35.4%	–	–

a Women were able to choose multiple responses to this question. The response options presented here include only the responses selected by at least one participant. The list provided was more expansive, including tampons, natural materials, no materials, and menstrual cups.

b Two women selected “choose not to answer.”

Over half of men (53%) reported that MWG in their household change materials in a room inside the house other than a bathing room or toilet, whereas only 37% of women said they changed materials in a room inside the house other than a bathing room or toilet. Women reported using a private toilet outside the house (20%), a private bathing room outside the house (21%), or a shared or public toilet (12%). Over a quarter of men (28%) chose “Don't know” when asked where MWG in their household change materials.

Men underestimated the extent to which women dispose of menstrual materials by flushing or putting them in a pit latrine. Specifically, only 3% of men reported that MWG in their household disposed of materials by putting them in toilets or latrines though 21% of women reported practicing this behavior. In addition, 12% of women reported that they do not dispose of menstrual materials, while only 1% of men said that MWG in their households do not dispose of menstrual materials. The most frequently reported disposal behavior was disposal in a rubbish bin (men: 49%, women: 58%). 35% of men selected “don't know” when asked about disposal.

Questions about whether or not it is appropriate for women to discuss menstruation were answered by 344 men and 297 women, as these were not restricted to those who menstruate or live with a MWG (Fig. 1). Half of men (50%) agreed that it is appropriate for women to discuss menstruation-related sanitation issues in front of men, compared to 30% of women (Table 3). There was a smaller difference when asked if it was appropriate for women to discuss menstruation-related sanitation issues publicly, with 30% of men and 24% of women agreeing with this statement (Table 3).

**Table 3:** Perception of norms surrounding discussion of menstruation.

Characteristic	Men N = 344		Women N = 297	
	N	%	N	%
**In this community, it is appropriate for women to discuss menstruation-related sanitation issues in front of men**^a^	335		297	
Agree	168	50.1%	88	29.6%
Disagree	167	50.0%	209	70.4%
**In this community, it is appropriate for women to discuss menstruation-related sanitation issues in public**^b^	336		297	
Agree	100	29.8%	72	24.2%
Disagree	236	70.2%	225	75.8%

a Nine men selected “don't know.”

b Eight men selected “don't know.”

### 3.3 Aim 2: associations between demographic characteristics and norms

In our assessment of the relationship between demographic characteristics and whether men agreed that discussing menstruation-related issues in front of men and publicly is appropriate, we found that men who lived with a MWG had a 1.37 times higher prevalence of reporting that it was acceptable for women to discuss menstruated-related issues in front of men (95% CI: 1.07, 1.76; p < 0.001) and a 1.66 times higher prevalence of acceptance of discussing menstruation-related issues publicly (95% CI: 1.12, 2.46; p < 0.01) (Table 4). Agreement that discussing menstruation in front of men was appropriate was 0.68 times as prevalent among men who identified as Baganda, the most common ethnic group in our sample, compared to men in all other ethnic groups (95% CI: 0.54, 0.85; p < 0.01). Similarly, agreement that it is appropriate for women to discuss menstruation publicly was 0.76 time higher in prevalence among Bagandan men (PR: 0.76; 95% CI: 0.53, 1.08; p = 0.13). Age, religion, and education did not have strong associations with agreement with either norm, as shown in Table 3. For age, which was treated as a continuous variable, the prevalence ratios indicate no relationship between age and discussing menstruation-related issues in front of men (PR:1.00; 95% CI: 0.99, 1.01; p = 0.84) and a 1% increase in prevalence of agreeing it's appropriate to discuss menstruation issues publicly for each one year of age.

**Table 4:** Poisson regression of household and demographic characteristics on prevalence of men's perceptions of norms around communicating about menstruation (prevalence ratio, 95% confidence interval, p-value).

Characteristic	Outcomes					
	It is appropriate for women to discuss menstruation-related issues in front of men.^a^ N = 308			It is appropriate for women to discuss menstruation-related issues publicly.∗ N = 310		
	PR	CI	P-value	PR	CI	P-value
**Presence of MWG in household**	1.37	1.07, 1.76	<0.001∗	1.66	1.12, 2.46	0.01^a^
**Age**	1.00	0.99, 1.01	0.84	1.01	0.99, 1.02	0.29
**Primary education or less**	0.88	0.63, 1.24	0.47	1.15	0.75, 1.77	0.51
**Religion: Christian (Catholic)**	1.03	0.81, 1.31	0.98	0.93	0.63, 1.35	0.69
**Religion: Muslim**	1.04	0.75, 1.44	0.80	0.82	0.47, 1.41	0.47
**Religion: Other**	1.00	0.60, 1.66	0.998	0.83	0.40, 1.74	0.62
**Ethnicity: Baganda**	0.68	0.54, 0.85	<0.01∗	0.76	0.53,1.08	0.13

∗P-value is less than 0.05.

a Adjusted for neighborhood.

### 3.4 Aim 3: associations between perception of norms and supportive behaviors

Our analyses of associations between men's perception of norms and meaningful support indicated that among men, compared to those who disagreed with both norms statement, agreeing with at least one norms statement was associated with 91% lower odds of reporting that women may be punished for speaking up about menstruation-related problems, compared to men who disagreed with both norms statements (OR: 0.09; 95% CI: 0.01, 0.37; p < 0.001) (Model 1a, Table 6). When we ran the model for men who agreed with both supportive norms statements, the odds ratio shrank slightly to 0.07 (95% CI: 0.00, 0.38; p = 0.01), indicating that agreeing with both norms statements was associated with 93% lower odds of reporting that women may be punished (Model 1b, Table 6).

**Table 5:** Reported supportive behaviors for MWG in household.^a^.

Reported behavior	Men N = 197	
	N	%
**Women in my household may be scolded or punished if they speak up about menstruation-related sanitation-concerns or problems**	195	
Agree	13	6.7%
Disagree	182	93.3%
**When women in my household face a problem related to menstruation and their sanitation-location, they can freely talk about the problem with me**^b^	193	
Yes	138	71.5%
No	55	28.5%
**I provide money for women in my household to meet their menstrual needs**^b^	193	
Agree	158	81.9%
Disagree	35	18.1%

a These questions were adapted to be specific to men with MWG in their household. The sample only includes men who live with a MWG.

b Two men selected “don't know.”

**Table 6:** Regressions of relationship between perceived norms and behaviors.

Characteristic	Outcomes								
	**Model 1a**^a^ **N=191**			**Model 2a** **N=189**			**Model 3a** **N=191**		

	Women in my household may be scolded or punished if they speak up about menstruation-related sanitation concerns or problems.			Women in my family can talk freely about problems related to menstruation with me.			I provide money for women in my household to meet their menstrual needs.		
	**OR**	**CI**	**P-value**	**PR**	**CI**	**P-value**	**PR**	**CI**	**P-value**
Agreement with at least one of the two norms	0.09	0.01, 0.37	**<0.001**∗	1.71	1.35, 2.16	**<0.001**∗	0.86	0.24,3.09	0.82

	**Model 1b**^a^ **N=151**			**Model 2b** **N=149**			**Model 3b** **N=151**		

Agreement with both norms	0.07	0.0004, 0.38	**0.001**∗	1.75	1.38, 2.22	**<0.001∗**	0.47	0.17,1.26	0.13

∗P-value is less than 0.05.

a Adjusted for neighborhood.

Men who reported at least one supportive norm had 1.71 times the prevalence (95% CI: 1.35, 2.16; p < 0.001) of agreeing that women in their family can speak about menstruation-related problems with them compared to men who disagreed with both norms statements (Model 2a, Table 6). Among men who reported agreement with both norms, there was a very small increase to 1.75 (95% CI: 2.22, 1.86; p < 0.001) (Model 2b, Table 6).

Men who agreed with at least one supportive norm had 0.86 times the prevalence of reporting that they provide money for women in their household to meet their menstrual needs, though the confidence interval for this estimate ranged from 0.24 to 3.09 (Model 3a, Table 3) indicating that this estimate may lack precision (p = 0.82). Men who agreed with both supportive norms had 0.47 times the prevalence of providing money for the menstrual needs of women in their household (95% CI: 0.17, 1.26, p = 0.13) (Model 3b, Table 3). The prevalence of providing money for menstrual needs was higher for men who only agree with one norm than for those who agree with two, though the small sample size for men who do *not* provide money for menstrual needs (n = 35, 18.1%) indicates that provision of money for menstrual needs is a widely-held practice (Table 5).

## 4 Discussion

In this study, we assessed alignment between women's reported menstruation practices and men's reports of menstruation practices of MWG in their household, identified associations between demographic characteristics and perceptions of supportive norms among men, and examined linkages between men's agreement with supportive norms and men's agreement with behaviors related to menstruation. Our analyses demonstrated gaps in men's understanding of women's actual menstrual practices, including what types of menstrual materials women used and women's menstrual material disposal practices. We found that having a MWG in the household was positively associated with supportive norms among men, while being of the Baganda ethnicity was associated with less supportive norms. Men's perception of supportive norms was positively associated with a willingness to talk about menstruation with women in their households and negatively associated with women being scolded or punished for speaking about menstruation. These results represent an important contribution to the literature by presenting data collected from adult men, a population largely unrepresented in menstruation-focused research, and by demonstrating that norms approving of open communication about menstruation are associated with more supportive behaviors.

The lack of alignment between the menstrual practices of women and what men reported has implications for women's health and well-being. Menstruation is a normal part of life from adolescence, and yet many men who live with menstruators are unaware of the realities of menstruation. Menstrual taboos, or norms that discourage speaking about menstruation, can be a barrier to seeking health-care advice when needed, can lead to shame and behavior restrictions, and may disrupt educational achievement or employment (Caruso et al., 2019; Wilson et al., 2018; Day, 2018). Menstrual secrecy therefore hinders gender equality, as it leads to negative impacts disproportionately borne by women and girls.

Men's lack of knowledge of women's practices may come in part from women's unwillingness to talk about menstruation in front of men. Our finding that women perceive less supportive norms around communication aligns with literature showing that women are often the ones to uphold menstrual secrecy and taboos (Mahon et al., 2015), that they perceive themselves to be violating social norms if they discuss menstruation with men and boys (Barrington et al., 2021), and that they often attempt to hide their menstrual waste or wash and dry menstrual cloths in private (Barrington et al., 2021; MacRae et al., 2019; Sommer et al., 2013; Caruso et al., 2017). However, this exclusion of men from conversations may be counterproductive to improving menstrual experiences. In one community water, sanitation, and hygiene intervention in India that sought to engage men in conversations around menstruation, men began to understand the importance of investing in supportive toilet infrastructure and budgeting for menstrual materials (Mahon et al., 2015).

Our results indicated that there was likely no relationship between supportive norms and provision of money to meet menstrual needs. There are a variety of possible explanations for this finding. 82% of men reported that they provide money for menstrual needs, and the parent study of this research found that in Kampala, 87% of women agreed or strongly agreed that they could independently make decisions about small sanitation-related purchases like soap or toilet paper. It is possible that women commonly control household budgets for regular household needs and sanitation (Sinharoy et al., 2024). Given that 70% of women in these communities believe that it is inappropriate for women to discuss menstruation in front of men, it is also possible that if men are providing money for household items, they are not aware of what amount is going specifically for menstrual materials but assume that the amount is sufficient for menstrual needs. This subject is worthy of further study; men's overestimates of how many women are using disposable pads and underestimates of how many women are using cloth could also mean that men are unaware of the cost of necessary purchases and women are going without needed materials or sacrificing other household items to purchase menstrual materials.

The gap between men's knowledge of women's practices and women's reported practices has important implications for sanitation infrastructure, particularly in urban environments like Kampala, where there is a reliance on shared or public toilets (Goddard and Marni, 2020). Disposal of single-use/disposable menstrual waste is ideally done in wastebins located near the toilet; disposal of menstrual products in toilets and pit latrines, a practice reported by 21% of women in our sample, can lead to clogging and blockages in sanitation systems and, in the absence of treatment facilities, become a source of a pollution (Sommer et al., 2013; Goddard and Marni, 2020). Re-using menstrual materials such as re-useable cloths and napkins, a practice underestimated by the men in our sample, requires the ability to wash and dry those materials in order to maintain gynecological health, meaning a source of water may be needed in sanitation locations (Das et al., 2015, 2021). In the case of both single-use/disposable and reusable materials, there are infrastructure requirements for women to deal with their menstrual products, and yet men may be unaware of those requirements and may fail to consult women in the design of toilets (Routray et al., 2017; Sommer et al., 2013). There is sufficient literature that demonstrates what is required for a toilet to be ‘female-friendly’ and supportive of menstrual health; however, men in leadership positions or making decisions about toilet infrastructure who may have limited engagement in discussions of menstruation may not realize the extent to which toilets are not meeting menstrual needs or what infrastructure is required (Hennegan et al., 2021; Schmitt et al., 2018). Norms stigmatizing menstruation may therefore not only harm women but may also harm local sanitation infrastructure.

The positive relationship we found between supportive norms and supportive behaviors and actions aligns with a large body of work that demonstrate how people's behaviors are influenced by the perception of what their peers and communities are doing across other contexts and activities (Borsari and Carey, 2003; Neighbors et al., 2010; Sood and Ramaiya, 2022; Mead et al., 2014). Our findings show decreased odds of women being punished or scolded for speaking up about menstruation in households where men perceive supportive norms. We also find a positive association between supportive norms and men's willingness to discuss menstruation problems. There are limited studies on norms around menstruation and men's behaviors; however, a school-based study in Tanzania found that among boys with statistically similar knowledge of biological facets of menstruation, there was an association between restrictive menstruation norms at home and period teasing (Benshaul-Tolonen et al., 2020). Addressing a lack of knowledge alone is insufficient for substantially improving the lives of menstruators if norms are not also addressed. A combination of education and norms change are needed to enable productive conversations around menstruation (Day, 2018).

There are considerable challenges in changing norms and addressing menstrual stigma (Olson et al., 2022; Bicchieri, 2016). However, norms change is a key avenue toward improving menstrual health (Hennegan et al., 2021; Day, 2018). Policies and interventions that aim to improve the lives of those who menstruate through menstrual health and education should pay special attention to dismantling norms of silence that discourage individuals who menstruate from discussing menstruation with those who do not. For example, KCCA has created menstruation working groups comprised of both men and women in an effort to normalize the inclusion of men in discussions around menstruation and to break menstrual stigmas; men have also been engaged in re-useable pad-making enterprises so men in households that lack the means to purchase single-use/disposable pads are equipped with methods to tangibly support MWG in their households. Programming should not neglect the importance of engaging non-menstruators, as they have considerable influence on the menstrual experiences of those who do menstruate.

## 5 Strengths and limitations

This study's inclusion of men in data collection about menstruation is an important contribution, given the limited research focused on men and menstruation. Because the ARISE scales were not validated among men, we were unable to calculate scores for men's overall perceptions of norms; however, we were able to use single prompts to explore specific norms (Sinharoy et al., 2022b). The parent study for this secondary analysis recruited men and women, and questions specifically ask about experiences and beliefs of men and women. This paper, therefore, does not capture experiences or beliefs of nonbinary or transgender individuals, which may differ from cisgender individuals, particularly in Uganda, where it is illegal to identify as LGBTQ+. (Human Rights, 2024) Because of challenges in recruiting all men and women from the same household, the data from men and women are not collected from household dyads. Therefore discrepancies in behaviors reported by men and women may not reflect an actual lack of knowledge about women's menstrual practices. However, because we conducted random sampling within neighborhoods, men and women are representative of the neighborhoods they live in. We do not have data on region of origin for those born outside Kampala, and therefore cannot assess the relationship between region of origin and menstruation norms. Additional research with larger sample sizes may result in more robust associations between norms and behaviors, and as this study is cross-sectional, we cannot show causality between perception of norms and behaviors. However, associations found here suggest that future work aiming to improve menstrual health should incorporate norms-change interventions into their planning.

Generalizability of study findings may be limited. We did not have a sampling frame that provided us with data about the overall populations in the specific neighborhoods engaged, which would have been useful to make comparisons with our sample. Further, we do not have data indicating the number individuals who were approached, who were eligible, or who consented, by gender, for each individual survey activity. That said, for all survey activities included in these neighborhoods, including the men and women's survey presented in this paper and the separate short-form survey with the same eligibility criteria delivered to women at the same time as part of the parent study, we do know that a high proportion of *all* individuals (both men and women) screened were eligible (884 individuals eligible out of 962 screened; 91.9%) to participate (69 out of 884 eligible participants did not consent; 7.8%). As such, while we are lacking some key data information, the high rates of eligibility and participation suggest that our sample is representative and that findings are generalizable for this study.

## 6 Conclusions

Our findings show that men play an important role in enabling menstrual health for women and girls who menstruate. As men often decide how household resources are allotted, they should be engaged in conversations about menstruation and educated on menstrual needs so that they can better support the menstruators in their lives through tangible means of creating a supportive physical environment, and intangible means of fostering a supportive social environment. Reducing shame and stigma surrounding menstruation will require the engagement of men and women, both separately and together.

## CRediT authorship contribution statement

**Madeleine Patrick:** Writing – review & editing, Writing – original draft, Visualization, Supervision, Project administration, Methodology, Data curation, Conceptualization. **Nicole Stephan:** Writing – review & editing, Writing – original draft, Visualization, Methodology, Formal analysis, Data curation. **Thea Mink:** Writing – review & editing, Data curation. **Tanushree Bhan:** Writing – review & editing. **Barbra Mary Aine:** Writing – review & editing, Methodology. **Nabutuwa Viola Matanda:** Writing – review & editing, Investigation. **Amelia Conrad:** Writing – review & editing, Methodology. **Sheela S. Sinharoy:** Writing – review & editing, Supervision, Project administration, Methodology, Funding acquisition, Conceptualization. **Bethany A. Caruso:** Writing – review & editing, Supervision, Project administration, Methodology, Funding acquisition, Conceptualization.

## Funding statement

This work was supported, in whole or in part, by the 10.13039/100000865Bill & Melinda Gates Foundation [Grant Number OPP1191625]. Under the grant conditions of the Foundation, a 10.13039/100026877Creative Commons Attribution 4.0 Generic License has already been assigned to the Author Accepted Manuscript version that might arise from this submission.

## Declaration of interests

The authors declare that they have no known competing financial interests or personal relationships that could have appeared to influence the work reported in this paper.

## Data Availability

Data are available on Figshare: [10.6084/m9.figshare.c.7590713].
