# Supplementary material for: Knowledge, attitudes, and support of women's menstrual experiences: A cross-sectional survey of men in Kampala, Uganda
Source: Health Place. 2025 May;93:103439. doi: 10.1016/j.healthplace.2025.103439 (PMC12064441; doi:10.1016/j.healthplace.2025.103439)
Supplement: Multimedia component 1 [file mmc1.docx]

**Supplementary Table A:** Regression *model structures, types, and sample sizes: unadjusted vs. adjusted for neighborhood effects*

| *Aim 2* | |
| --- | --- |
| *A. Models of association between men’s demographic characteristics, presence of a menstruating woman or girl in home (MWG), and* ***men’s perception of norms around speaking about menstruation in front of men***  *(Poisson regression, n=308* | |
| A1. MWG + age + education + religion + ethnicity | **A2. MWG + age + education + religion + ethnicity + neighborhood** |
| *B. Models of association between men’s demographic characteristics, presence of a menstruating woman or girl in home (MWG), and* ***men’s perception of norms around speaking about menstruation in public***  *(Poisson regression n=310* | |
| B1. MWG + age + education + religion + ethnicity | **B2. MWG + age + education + religion + ethnicity + neighborhood** |
| *Aim 3* | |
| *C. Models of association between men who agree with at least one norm around speaking about menstruation and who live in households where* ***women who speak up about menstruation-related sanitation concerns or problems may be punished or scolded***  *(Firth’s logistic regression, n=191* | |
| C1. Men agreeing with at least one norm | **C2. Men agreeing with at least one norm + neighborhood** |
| *D. Models of association between men who agree with both norms around speaking about menstruation and who live in households where* ***women who speak up about menstruation-related sanitation concerns or problems may be punished or scolded***  *(Firth's logistic regression, n=151* | |
| D1. Men agreeing with both norms | **D2. Men agreeing with both norms + neighborhood** |
| *E. Models of association between men who agree with at least one norm around speaking about menstruation and are* ***willing to talk to women in their household about menstruation problems***  *(Poisson regression, n=189* | |
| **E1. Men agreeing with at least one norm** | E2. Men agreeing with at least one norm + neighborhood |
| *F. Models of association between men who agree with both norms around speaking about menstruation and are* ***willing to talk to women in their household about menstruation problems***  *Poisson regression, n=149* | |
| **F1. Men agreeing with both norms** | F2. Men agreeing with both norms + neighborhood |
| *G. Models of association between men who agree with at least one norm around speaking about menstruation and* ***provide money for women to meet menstruation needs***  *Poisson regression, n=191* | |
| **G1. Men agreeing with at least one norm** | G2. Men agreeing with at least one norm + neighborhood |
| *H. Models of association between men who agree with both norms around speaking about menstruation and* ***provide money for women to meet menstruation needs***  *Poisson regression, n=151* | |
| **H1. Men agreeing with both norms** | H2. Men agreeing with both norms + neighborhood |

**Bolded text indicates the model that was used in final analyses based on confounding assessment**

**Supplementary Table B**

| **Logistic regression of household and demographic characteristics on prevalence of men’s perceptions of norms around communicating about menstruation (prevalence ratio, confidence interval, p-value)** | | | | | | | | | | | | |
| --- | --- | --- | --- | --- | --- | --- | --- | --- | --- | --- | --- | --- |
|  | **Unadjusted Outcomes** | | | | | |  | **Adjusted Outcomes** | | | | |
| **Characteristic** | **It is appropriate for women to discuss menstruation-related issues in front of men.**  **N=308** | | | **It is appropriate for women to discuss menstruation-related issues publicly.**  **N=310** | | | **It is appropriate for women to discuss menstruation-related issues in front of men. N=308** | | | **It is appropriate for women to discuss menstruation-related issues publicly.**  **N=310** | | |
|  | **PR** | **CI** | **P-value** | **PR** | **CI** | **P-value** | **PR** | **CI** | **P-value** | **PR** | **CI** | **P-value** |
| **Presence of MWG in household** | 0.53 | 0.37, 0.76 | <0.001* | 0.21 | 0.12, 0.37 | <0.001* | 1.37 | 1.07, 1.76 | <.001* | 1.66 | 1.12, 2.46 | 0.01* |
| **Age** | 1.40 | 1.09, 1.80 | 0.01* | 1.75 | 1.16, 2.63 | 0.01* | 1.00 | 0.99, 1.01 | 0.84 | 1.01 | 0.99, 1.02 | 0.29 |
| **Primary education or less** | 1.00 | 0.99, 1.01 | 0.77 | 1.01 | 0.99, 1.02 | 0.40 | 0.88 | 0.63, 1.24 | 0.47 | 1.15 | 0.75, 1.77 | 0.51 |
| **Religion: Christian (Catholic)** | 0.87 | 0.62, 1.23 | 0.44 | 1.11 | 0.72, 1.72 | 0.62 | 1.03 | 0.81, 1.31 | 0.98 | 0.93 | 0.63, 1.35 | 0.69 |
| **Religion: Muslim** | 1.00 | 0.79, 1.26 | 0.98 | 0.81 | 0.56, 1.19 | 0.28 | 1.04 | 0.75, 1.44 | 0.80 | 0.82 | 0.47, 1.41 | 0.47 |
| **Religion: Other** | 0.98 | 0.58, 1.66 | 0.95 | 0.83 | 0.47, 1.44 | 0.50 | 1.00 | 0.60, 1.66 | 0.998 | 0.83 | 0.40, 1.74 | 0.62 |
| **Ethnicity: Baganda** | 1.04 | 0.75, 1.45 | 0.81 | 0.81 | 0.35, 1.87 | 0.62 | 0.68 | 0.54, 0.85 | 0.001* | 0.76 | 0.53,1.08 | 0.13 |
| *P-value is less than 0.05, indicating statistical significance | | | | | | |  |  |  |  |  |  |

**Supplementary Table C**

| **Regressions of relationship between perceived norms and behaviors** | | | | | | | | | |
| --- | --- | --- | --- | --- | --- | --- | --- | --- | --- |
| **Characteristic** | **Outcomes** | | | | | | | | |
|  | **Model 1a**  **N=191** | | | **Model 2a**  **N=189** | | | **Model 3a**  **N=191** | | |
|  | Women in my household may be scolded or punished if they speak up about menstruation-related sanitation concerns or problems. | | | Women in my family can talk freely about problems related to menstruation with me. | | | I provide money for women in my household to meet their menstrual needs. | | |
|  | **OR** | **CI** | **P-value** | **PR** | **CI** | **P-value** | **PR** | **CI** | **P-value** |
| Agreement with at least one of the two norms (unadjusted) | 0.07 | 0.01, 0.32 | **<0.001*** | 1.71 | 1.35, 2.16 | **<0.001*** | 0.86 | 0.24,3.09 | 0.82 |
| Agreement with at least one of the two norms (adjusted for neighborhood) | 0.09 | 0.01, 0.37 | **<0.001*** | 1.72 | 1.36, 2.16 | **<0.001*** | 0.89 | 0.26, 3.08 | 0.86 |
|  | **Model 1b**  **N=151** | | | **Model 2b**  **N=149** | | | **Model 3b**  **N=151** | | |
| Agreement with both norms (unadjusted) | 0.04 | 0.0003, 0.30 | **0.003** | 1.75 | 1.38, 2.22 | **<0.001*** | 0.47 | 0.17,1.26 | 0.13 |
| Agreement with both norms (adjusted for neighborhood) | 0.07 | 0.0004, 0.38 | **0.001*** | 1.80 | 1.42, 2.27 | **<0.001*** | 0.49 | 0.19, 1.22 | 0.19 |
| *P-value is less than 0.05, indicating statistical significance | | | | | | | | | |
